# Challenges and Opportunities for Clinical Cytogenetics in the 21st Century

**DOI:** 10.3390/genes14020493

**Published:** 2023-02-15

**Authors:** Eric Heng, Sanjana Thanedar, Henry H. Heng

**Affiliations:** 1 Stanford University, 450 Jane Stanford Way, Stanford, CA 94305, USA; 2 Molecular Medicine and Genetics, Wayne State University School of Medicine, Detroit, MI 48201, USA; 3 Department of Pathology, Wayne State University School of Medicine, Detroit, MI 48201, USA

**Keywords:** chromosome instability (CIN), chromosomics, cytogenomics, FISH, genome instability, heteromorphism, karyotype coding, mosaicism, non-clonal chromosome aberrations (NCCAs), two-phased cancer evolution

## Abstract

The powerful utilities of current DNA sequencing technology question the value of developing clinical cytogenetics any further. By briefly reviewing the historical and current challenges of cytogenetics, the new conceptual and technological platform of the 21st century clinical cytogenetics is presented. Particularly, the genome architecture theory (GAT) has been used as a new framework to emphasize the importance of clinical cytogenetics in the genomic era, as karyotype dynamics play a central role in information-based genomics and genome-based macroevolution. Furthermore, many diseases can be linked to elevated levels of genomic variations within a given environment. With karyotype coding in mind, new opportunities for clinical cytogenetics are discussed to integrate genomics back into cytogenetics, as karyotypic context represents a new type of genomic information that organizes gene interactions. The proposed research frontiers include: 1. focusing on karyotypic heterogeneity (e.g., classifying non-clonal chromosome aberrations (NCCAs), studying mosaicism, heteromorphism, and nuclear architecture alteration-mediated diseases), 2. monitoring the process of somatic evolution by characterizing genome instability and illustrating the relationship between stress, karyotype dynamics, and diseases, and 3. developing methods to integrate genomic data and cytogenomics. We hope that these perspectives can trigger further discussion beyond traditional chromosomal analyses. Future clinical cytogenetics should profile chromosome instability-mediated somatic evolution, as well as the degree of non-clonal chromosomal aberrations that monitor the genomic system’s stress response. Using this platform, many common and complex disease conditions, including the aging process, can be effectively and tangibly monitored for health benefits.

## 1. Introduction

The main goal of clinical cytogenetics is to reveal the relationship between chromosomal/nuclear alterations and various genetic conditions related to human health. This medical discipline mainly studies human pathogenic chromosomal abnormalities, which can be used for patient management including diagnosis, prognosis, treatment, and genetic counseling. Historically, clinical cytogenetics has helped the identification of many genes that contribute to various inheritable diseases. Even though clinical cytogenetics is a subset of cytogenetics, it has occasionally been at the forefront of the entire field due to advanced visual technology, a diverse range of clinical samples, better funding, and the scientists’ fascination with their chromosomes. That is why the identification of the correct number of human chromosomes represents such a milestone [1]. Similarly, the discovery of the chromosomal basis for Down’s syndrome [2], the identification of the Philadelphia chromosome for Chronic Myeloid Leukemia (CML) [3,4], and the establishment of databases of chromosome aberrations and diseases played an influential role in the direction of genetic disease research [5].

As soon as biological research entered the molecular era in the 1950s (marked by the DNA double helix model), particularly following the success of molecular cloning in the 1970s (marked by recombinant DNA technology), clinical genetics was dominated by a gene-centric perspective, and discussions about replacing cytogenetic analysis with gene profiles sometimes came up. The rationale for replacing cytogenetics with molecular genetics seems logical: cytogenetics had a supporting role in genetics as chromosomes were seen as simply carriers of genes. According to reductionist viewpoints, higher sensitivity and resolution are directly correlated with mechanistic understanding [6]. In addition, cytogenetic analyses are time-consuming and banding pattern identification could be subjective (molecular probes increased the accuracy of cytogenetic approaches later). Chromosome preparation often requires access to mitotic figures, which excluded non-dividing cell populations. Moreover, cytogenetics processes, unlike traditional molecular biology techniques, are hard to fully automate: while sequencing analysis can be done with a machine, analyzing a slide of chromosomes has not yet been mechanized. Finally, with the drastic reduction in sequencing costs, why should cytogenetic analyses be used? The “sequence everything” attitude seemed to have been the last nail in the coffin for cytogenetics. With the retirement of many cytogenetic scholars, the new generation of researchers is no longer attracted by clinical cytogenetics as a profession, and even directors of medical cytogenetic laboratories in the United States are eagerly obtaining extra board certification in genomics, anticipating that the change will soon come.

Historically, cytogenetics has evolved as a unique discipline in response to the stagnation of the field; new cytogenetic techniques and discoveries generally take place just when some start to question the value of the field. Prior to the discovery of hypotonic treatment and air-dry methods, for example, cytogeneticists preferred dealing with plant chromosomes over human chromosomes due to the technical difficulty of preparing chromosome spreads [7]. This series of technological innovations also promoted the establishment of clinical cytogenetics. In the late 60s, just when the classical comparison of the number and size of chromosomes seemed to reach a technical ceiling, chromosomal banding was born [8,9]. Various banding methods drastically advanced cytogenetics, linking specific chromosomes and regions to particular diseases. In the middle of disease gene hunting, chromosomal mapping become popular; meanwhile, the chromosomal identification of translocation regions played an important role in cloning fusion genes. In the golden age of molecular genetics, emergent molecular cytogenetics, armed with diverse fluorescence in situ hybridization (FISH) technologies, greatly contributed to medical genetics and physical mapping, an initial phase of the human genome project [10]. These molecular cytogenetic approaches included FISH on banded chromosomes, fiber FISH, SKY (Spectral karyotype)/M-FISH, FISH-derived CGH, DNA-protein in situ co-detection, helo-FISH, 3D-FISH, and Q-FISH, to name a few [11,12,13,14,15,16,17,18,19,20]. The combination of these methods with gene characterization and cellular functional analyses extended the linkage of chromatin domain behavior to diseases and the function of various genes for maintaining mitotic, meiotic, and embryogenic processes, revealing the highly dynamic cancer genome [21,22,23]. In the current large scale of the omics era, various array methods, such as the single-nucleotide polymorphism array and the Comparative Genomic Hybridization array, become an essential component of medical cytogenetic platforms [23,24,25]. With the further transition from genetics to genomics [6], coupled with the continuous blurring of the boundaries between molecular biology and molecular cytogenetics [26], there has been an increased acceptance of the suggested transition from cytogenetics to cytogenomics, which would include the profiling of copy number variation and sequence-converted data. While opinions differ on the use of the term ‘cytogenomics’ [27], it is not yet clear what advantages cytogenetics has in the era of sequencing. However, if history can tell us anything, novel powerful technologies should be what we are looking for. 

Surprisingly, while current large-scale omics projects have generated massive amounts of molecular data, they have also unexpectedly revealed the key limitations of the gene-centric genetic theory [6,28]. In particular, the huge gap between genotypes and phenotypes cannot be bridged by the increased knowledge of the genes [6,29,30]. This division requires a search for different levels of genomic organization that are responsible for organizing genetic networks and managing highly heterogeneous information in the many codes found in the genome, from the genetic code, to the chromatin organization code, to the genomic topology code [31]. Thus, instead of looking for the answers in the genes, we should be looking for answers in the systems that organize the genes—in chromosomes, for example. Thus, a better paradigm is needed to redefine and then unify genotype and phenotype. Continuing to search for DNA sequencing data using the same gene-centered approach that has not produced desired results seems like a fruitless endeavor.

In mid-2000s, cytogenetics renewed its tradition of studying inheritance above the gene level and even the role of chromosomes in controlling genes (not just the carrier of gene master). Such a tradition was established by pioneers including Richard Goldschmidt and Barbara McClintock [32,33]. Equipped with molecular probes, spatial cytogenetics was popularized (far earlier than current high-C and single-cell sequence-promoted spatial biology) [34,35]. Based on methods of visualizing different chromatin domains with different colors, the spatial relationship of chromatin behavior in the nucleus has been studied. Such analyses link chromatin types and distributions with nuclear matrices and gene expression, illustrate the AT/GC content of specific sequences and the chromosomal positions that impact loop size and function, function as a platform to study genes’ genomic environment, and reveal the potential contribution of chromatin to diseases [36,37]. This exciting development has led Professor Uwe Claussen (Jena, Germany) to propose the term cytogenomics to describe the new cytogenetics [38]. According to Thomas Liehr, “(Claussen) suggested to introduce the term chromosomics being equal to cytogenomics to bring the three-dimensional morphologically of chromosomes into the focus of research, as this is essential for gene regulation. Under this generic term, all chromosome-related studies should be summarized to introduce novel ideas and concepts in biology and medicine, thus having an integrative effect on the field” [27]. Clearly, introducing cytogenomics represents an important effort to redefine the framework of new cytogenetics. Interestingly, in Claussen’s 2005 paper, he referred to our article “Re-defining the chromatin loop domain” [39] as “a good starting signal for chromosomics”. Indeed, from 1994 to 2004, we have pushed for studying the dynamics of the chromatin loop and its constraints on genes’ function, which further challenged the gene-centric concept. More significantly, since the 2000s, cancer cytogenetic research has led the effort to redefine cancer evolution, the relationship between genes and chromosomes, and the new conceptual frameworks of 21st century cytogenetics and cytogenomics. By profiling both multiple levels of genotypes (gene, transcriptome, and karyotype) and phenotypes (individual cell and population dynamics) during the cancer evolutionary process, it is obvious that the cytogenetic profile plays a leading role in macro-cellular evolution. Furthermore, the understanding of the relationship between clonal chromosome aberrations (CCAs) and non-clonal chromosome aberrations (NCCAs) holds the key for studying and integrating phase transitions in cellular evolution, genomic information management, stress and response-mediated karyotype heterogeneity, and their relationship with human diseases. By linking system information to karyotype coding and genome type variations, we not only can better understand the meaning of the cytogenetic data in a holistic systems perspective but also should apply this new knowledge to clinical cytogenetics, as cellular evolution is the common basis for many human diseases [40].

Altogether, the ultimate importance of cytogenetics becomes obvious, which nicely explains, and further extends, the vision of many visionaries, including Barbara McClintock, and solves puzzles in genomics and evolution, as well as providing new cytogenetic platforms for disease research and clinical usage.

## 2. The New Cytogenetic Framework in the Era of Large-Scale Genomics and System Biology

Even though cytogenetic technologies have from time to time occupied the forefront of genetics [41], cytogenetics rarely contributes to a basic genomic framework that unifies genetics and genomics. In her 1983 Nobel Prize acceptance lecture, Barbara McClintock pointed out the ultimate importance of studying genomes rather than genes under stress in future biology [42]. Unfortunately, due to the domination of gene-centric thinking, her message was largely ignored. By watching cancer evolution in action, we have systematically traced genome-level evolution generation by generation. Following over two decades of research and synthesis, the Genome Architecture Theory (also called the Genome Theory) has been proposed to serve as a genome- and information-based conceptual framework for genomics and evolution. Table 1 briefly summarizes some highlights of this new theory which can further advance various subfields of genetics, genomics, and system biology including clinical cytogenetics.

As illustrated in Table 1, the genome- and information-based theoretical framework can solve many confusions and unify different ideas. For instance, determining the genomic basis for a species’ genetic networks has been a puzzle. Now, karyotype coding provides the basis for a more detailed illustration. The restored importance of the karyotype will also be welcomed by the cytogenetic community.

## 3. New Opportunities for Cytogenetics and Cytogenomics 

While important, the proposed genome architecture theory (GAT) needs to be further developed (with combinational details involving genes, chromosomes, and epigenetic mechanisms) and validated. More importantly, this alternative concept should further provide novel and unique utilities for clinical cytogenetic analyses.

### 3.1. Studying Non-Clonal Chromosome Aberrations and Multiple Types of Cyto-Heterogeneity

#### 3.1.1. Collection of NCCAs to Monitor the Genome Instability (Both Inherited and Induced Types)

NCCAs have often been ignored in clinical cytogenetic analyses as they were considered insignificant “noise” [64], even though increased frequencies of NCCAs have been associated with drug resistance and poor prognoses in cancer. In addition, the uncertainty of NCCAs has made them hard to study, especially when their molecular mechanisms are unknown. A series of studies have also linked the frequencies of NCCAs to chromosome instability (CIN) [46,47], and the existence of NCCAs is likely contributed to by the dynamics of inheritance such as fuzzy inheritance [43]. The non-linear relationship between NCCAs and CCAs has revealed the pattern of genome-mediated cancer evolution, which suggests that the two-phase cancer evolution is non-Darwinian evolution [53]. In recent years, increasing numbers of researchers have applied the concept of NCCAs for data analyses, reflecting the initial acceptance of profiling genome-level heterogeneity in various disease conditions [65,66,67,68,69,70,71,72,73,74]. Here, we call for the action of reporting NCCAs as well. For both NCCAs and CCAs, chromosomal profiles are more meaningful when considering CIN as an important factor for cancer patients.

#### 3.1.2. Identification and Classification of New Types of Chromosome/Nuclei Variations

Before using NCCAs in clinical cytogenetics, systematic validations are needed. First, it is necessary to study all types of NCCAs, including many previously unreported ones [74]. The comparison and classification among various types of NCCAs, including nuclear aberrations, represent an important step in applying NCCAs to clinical cytogenetics. Equally important is to illustrate if certain types of NCCAs can be associated with specific types of diseases.

In recent years, various subtypes of chaotic genomes have been reported, including chromothripsis, chromoplexy, polyploid giant cancer cell, and micronuclei clusters [55,56,57,58,59,60,61,62,63,75,76,77,78]. Traditionally, most chaotic karyotypes are ignored based on the assumption that these cells will be eliminated. Now, it is clear that though the majority of these drastically altered genomes will be eliminated, it is almost certain that some of them will survive and play an important role in producing aggressive cell populations. Such “unlucky” outliers can lead to the emergence of new cell populations, which represent a powerful mechanism for cancer evolution. The common mechanism is the formation of new genome systems via genome reorganization by altering karyotype coding under crisis. These extreme forms of NCCAs are key for the transition between macro- and microevolution.

#### 3.1.3. Mosaicism

Starting from the last decade, somatic genomic mosaicism has become a hot topic in genomic research [79]. One of the big surprises of current genomics is the realization that genomic variations are more common and impactful than previously believed. One form of this variation is mosaicism. From earlier development to the aging process, the high level of genomic heterogeneity is of importance for somatic adaptation and is not simply caused by ”mistakes”. Instead, high levels of heterogeneity are favored by organisms as an active strategy as well. As the result, all humans are composed of genetic mosaics, and the extent of this mosaicism can provide insight into both their evolutionary history and the level of stress they have experienced [80,81]. The mechanism of mosaics can be referred to as “fuzzy inheritance”, where inheritance is defined by a spectrum of possible phenotypes [6]. The karyotypic mosaics have been discussed in Down’s syndrome [82], various neurodevelopmental/neurobehavioral and neuropsychiatric disorders, neurodegeneration, cancer, and aging [83,84,85]. Phenotypic variations, including treatment response, are likely contributed to by the degree of mosaicism, and we anticipate that more diseases will be linked to somatic genomic mosaicism soon.

#### 3.1.4. Polymorphism

Heteromorphism or chromosomal polymorphism has been studied in clinical cytogenetics for decades. For example, research interests have involved the heterochromatin regions of chromosomes 1, 9, 16, Y, prominent acrocentric short arms, and satellites in the past decades [86]. Though some consider chromosomal polymorphisms a normal variant, others insist that these variants should not be ignored in clinical cytogenetic analyses. Furthermore, though the mechanism of this phenomenon is not fully understood, the enrichment of repetitive sequences and transposable elements has definitively been implicated. To date, association studies have been performed to study their relationship with reproductive impact, but further studies are needed to increase confidence [87,88]. A recent article revisited the significance of studying chromosomal heteromorphisms in disease by focusing on the example of cancer. In this analysis, many interesting aspects of heteromorphism were covered, including its relationship to cytogenetically visible copy number variations (CG-CNVs), size variants of the centromeres, euchromatic variants, and lncRNA. Overall, chromosomal polymorphism plays a role in disease development and/or susceptibility [89]. Based on the genome architecture theory (GAT), the increased number of variables, including chromosomal levels of polymorphism, can be increased if overall system instability is high, and the consequences could be good, neutral, or bad depending on the overall genomic landscape and its environmental interaction. The observed association of polymorphism and aneuploidy support such a prediction. Future research should examine if additional specific chromosomal changes or the overall genome instability play any role in disease phenotypes, as the combinational variables can also be understood as the emergent properties of diseases.

Moreover, since “all sequences used in molecular cytogenetic routine diagnostics to detect heterochromatic and/ or pericentromeric satellite DNA sequences within the human genome are not included yet into human reference genome” [90], cytogenetic analyses on heteromorphisms will not be replaced by DNA analysis soon.

#### 3.1.5. Nuclear Architecture and Diseases

Nuclear architecture, including the distribution of chromatin domains, individual chromosomes, telomeres, and centromeres, has long been studied in cytogenetics, especially promoted by multiple color-FISH and 3D image technologies [16,39,91]. With the establishment of the chromosome territories concept [92,93], chromatin structure/function/behavior has been linked to gene expression dynamics, the AT or GC-rich regions, the integrity of the genome, the location of the chromosomes, the mechanism of chromosomal/nuclear reorganization, and disease phenotype [13,17,94,95].

As soon as the topological distribution of chromosomes 9 and 22 in cell nuclei was linked to the induction of t(9;22) translocations in leukemias [96], similar studies have supported the conclusion that the formation of specific translocations in lymphomas, and likely other tissues, is determined in part by the chromosome topological relationship within the genome [97,98]. Recently, using structured illumination microscopy and 3D reconstruction, the large-scale topological disruption of chromosome territories 9 and 22 has been linked with nonresponse to treatment in CML, suggesting yet another mechanism of drug resistance [99]. Notably, other variables including the behavior of telomeres, the impact of structural variations, micronuclei clusters, and even cancer genes can be linked to disease via nuclear architecture. A similar analysis can be applied to other types of diseases.

Interestingly, despite the popularity of high-C technology, cytogenetic analysis represents an important validation. Furthermore, the cytogenetic platform can easily provide information that can be extrapolated from a single cell to a population, which is important to study the relationship between the average profile of a population and outliers.

### 3.2. Monitoring the Process of Somatic Evolution under Various Physiological, Pathological, and Medical Conditions

Most diseases have their developmental or genomic/epigenetic basis that ultimately interacts with environments during somatic evolution. One important evolutionary genomic feature of the disease evolution is the dynamics of NCCAs, which can be used for health management including diagnosis, treatment management, and prediction.

#### 3.2.1. Monitor Individualized Genome Instability during Disease Evolution

Since NCCA has been linked to genome instability that can significantly contribute to disease [6,35,40,74,78], examining inherited and induced genome instability should be used for studying many types of common and complex diseases. Furthermore, as disease evolution is a process, longitudinal profiling is necessary to understand the disease procession and possibly the treatment options. For example, it is possible to classify cancer patients into different categories based on an individual’s genome instability. For those who display a high level of NCCAs (thus, a high degree of genome instability), the aggressive drug treatment could trigger genome chaos leading to rapid drug resistance [53,55,56]. Even for the same individual, the treatment option will be influenced by the phase of cancer evolution. Finally, even the treatment responses and prognosis are different among patients with different genome instability. Interestingly, similar approaches can be applied to many non-cancer types of diseases [40,85,100], even though most examples are from cancer research. Therefore, systematically monitoring the genome instability with cytogenetic tools should be commonly used in the future.

#### 3.2.2. Studying Genome Instability in Developmental and Aging Processes

One surprising observation of earlier human development is the involvement of a highly unstable genome or genome chaos [6]. Recently, this phenomenon has been studied for understanding the PGCCs (Polyploidy Giant Cancer Cells) in the context of development and somatic macroevolution [49,57,58,59,60,61]. One interesting question is to study the mechanism of how embryo development overcomes genome chaos, which can offer insights into fighting cancer. The cytogenetic analysis combined with cellular lineage tracing will contribute to this effect. From a clinical cytogenetic perspective, studying some genome outliers will be valuable not just for monitoring in vitro fertilization, but diseases involving earlier development.

Aging symbolizes a different kind of life phenomenon in contrast to earlier development. Interestingly, one key feature of aging is the increased instability reflected by increased NCCAs. Amongst these many layers of genomic and nongenomic (including epigenetic) instabilities that contribute to aging [83,84], genome instability is ultimately important. To match the increased medical intervention on aging, a cytogenetic monitoring system is needed to trace and predict the aging process and results of aging interventions.

#### 3.2.3. Studying Environment-Contributed Diseases

Inheritable diseases with a definite correlation with abnormal patterns of karyotype and/or CNVs have traditionally been examined in clinical cytogenetics. Because of this, prenatal and cancer diagnostic analyses represent the main body of literature. For many common and complex diseases, however, the specific genomic pattern is hard to identify due to many factors involved, the nature of heterogeneity, and the overall environmental contribution, which is often more dominant than specific genetic factors. Nevertheless, the important linkage of CIN and experimental stress response, as well as disease status, provides a new platform to study genomic contributions to many diseases via environmental-induced genome instability [6,54,101,102]. Specifically, the impact of lifestyle, including nutrition, and working environments can be studied simply based on the dynamics of NCCA-mediated CIN. The comparison can be further extended into the similarity and difference between chronic vs. acute stress and damage, and specific and non-specific system response and evolution. This new platform can effectively study different types of common and complex diseases. All this information can play a role in guiding research and practice.

#### 3.2.4. Monitoring the Dynamic Impact of Medical Treatment

This category belongs to a special type of environmental impact on human health. Due to its importance, it is necessary to discuss this issue as an independent topic. As an unavoidable trade-off, any medical intervention can harm some patients under certain conditions. With the advancement of molecular medicine, some interventions become very powerful in targeting molecular pathways but could unexpectedly damage the whole system. Sometimes, the short-term benefits can turn into long-term negative results as well when used improperly. Such transition is linked to stress-induced system behavior. In complex systems, targeting lower levels may have unexpected results on higher levels, while targeting a particular molecule may also result in a non-specific reaction. Therefore, monitoring the system behavior is important for designing results and reducing the negative impact. Using NCCAs to monitor the medical-treatment-induced system instability is important, as high levels of instability can often generate opposite results. For example, the treatment-induced genome chaos can lead to rapid and massive drug resistance in cancer [49,53,56,58,59]; targeting the level of a specific “bad” molecule can generate new stress in the system to harm some patients [103]; stem cell reprogramming can sometimes lead to cancer; and gene modification including gene editing by CRISPR/Cas9 can trigger the genome alterations leading to macroevolution [6,104,105,106]. To safely apply all these medical interventions, these procedures should be carefully monitored by clinical cytogenetic methods (not to trigger genome chaos, for example). Similar tests should be used for evaluating new drugs before using them on patients.

It should be noted that to monitor the somatic evolutionary process effectively, ecological features at the cell and tissue level must be considered. That is the reason why spatial biology and topology in biology are important. Over a decade ago, we proposed the use of a 4D genomic platform to combine cytogenetics with evolution [107]. Five years ago, spatial transcriptomics technologies began development, making it possible to put 4D genomics into practice. In the future, more powerful methods will be developed based on a combination of cytogenetics and molecular biology, including sensitive in situ hybridization, in situ sequencing, spatial transcriptomics, topological informatics, and cellular information management. Combinational platforms, including those that are yet to be developed, hold the key to the future of clinical cytogenetics. Although karyotype information is crucial, new technologies that profile karyotypes at various levels of resolution have the potential to revolutionize the field even further. Clearly, a new revolution in understanding health and disease is emerging [108].

### 3.3. New Methods Development

The key development of molecular cytogenetics in the past few decades has been the capability of blurring the boundaries between cytogenetics and molecular biology [26]. To face today’s challenges, more combinational methods of cytogenetics and genomics are needed to advance molecular cytogenetics and cytogenomics. The following frontiers desire more attention.

#### 3.3.1. Establishing Data Analysis Platforms to Convert DNA Sequencing Data into Cytogenetic/Genomic Data

It is shown that cytogenetic data (karyotype and/or CNVs) have a better clinical prediction value than DNA data [43,44,45], supporting the evolutionary power of the genome system rather than the parts of genes. This type of data conversion can actually provide the genomic and system context for sequence data. For example, we can effectively convert DNA sequence data into karyotype data so that CIN can be measured. The CIN data can then unify different individual molecular mechanisms. Recently, bioinformatics platforms were introduced to convert sequence data into chromosomal data [109]. The platforms for using DNA data (such as array comparative genomic hybridization (arrayCGH) and SNP arrays) for generating high-resolution karyotypes in silico have also emerged. Equally important, analytical programs are needed to use cytogenetic data for disease research. It was illustrated that the endpoints of chromosome rearrangement alone can predict cancer types with more reliability and specificity [110]. Recently, an effort has also been made to quantify CIN [111,112].

It should be pointed out that, even though the costs of single-cell sequences are decreasing drastically, it can be costly to study the heterogeneity issue when some outliers are at the rate of 1% or lower. In contrast, scoring specific NCCAs among a few hundred mitotic figures can be effectively achieved. In this case, molecular cytogenetic tools are better than repeating sequencing hundreds of times.

#### 3.3.2. Comparing the Capability of Monitoring Genome Instability Using Different Types of Non-Clonal Chromosome Aberrations

Currently, the total frequencies of structural and some numerical NCCAs are used to monitor the CIN. However, some types of NCCAs may play a more dominant role than other types. For example, chromosomal translocation and polyploidy are more dominant than aneuploidy for predicting tumorigenesis [22], and the chaotic genome with many multiple translocations in one mitotic cell contributes more than a cell with a single translocation. With the increased types of NCCAs being characterized, and more diseases being linked to NCCAs, some types of NCCAs may be more frequently detected from certain types of diseases. Alternatively, it is also possible that only the quantitative NCCAs matter the most. Only systematical comparison will tell.

#### 3.3.3. Establish NCCAs Database

To effectively use NCCAs in clinical cytogenetics, it is essential to establish the baseline of different types of NCCAs in healthy individuals and the increased frequencies in various diseases and/or medical treatments. As different types of NCCAs can be linked through the cell cycle, where DNA replication, chromosome condensation, chromosomal segregation, and decondensation are ultimately related key steps [113,114], basic research is also required to study the relationship between various NCCA types. In fact, the defective mitotic figures or DMFs were among the initial studied NCCAs, and the condensation errors have been linked to DNA replication error and aneuploidy, chromosome fragmentations, and genome chaos [6,113,115,116,117]. Similarly, the micronuclei and their clusters represent another example of NCCA study, which can be linked to many biological processes [78,85]. Regardless of the types of NCCAs, they all can be understood as an active means to create or modify genomic information essential for either cellular adaptation and/or survival [49], in addition to functioning as the result of passive system errors/damage under stress.

The next step is to establish databases of NCCAs and diseases. Interestingly, some researchers have kept the NCCA data for decades without publishing them (Rowley J, personal communication). It is time to collect NCCA data, publish them, and organize them with an easy-to-access database. All these “dark matter” types of variants need to be understood.

This database should also include the NCCA baseline of different ethnic groups, as a given population’s genomic profile can influence the explanation of individuals’ profiles. For example, it is suggested that CNVs’ analysis must be carried out using an ethnically appropriate reference population [118,119].

#### 3.3.4. Further Improvement and Implementation of Cytogenetic Platforms in Clinical Cytogenetics

Despite the importance of karyotype, the field needs the new capability to integrate genomic data for better clinical usage. One approach is the standardization of combinational analysis of classical karyotype analysis with CNVs, various arrays, and other molecular cytogenetic data, particularly, as we discussed earlier, significantly improving the capability of converting sequencing data into a digital karyotype. Active collaboration might be needed with AI technology. Another strategy further improves visual methods, which can directly “see” the genotypes. For example, the direct observation of the common pattern of NCCAs and the chaotic genome during cancer macroevolution, including PGCCs during drug resistance, has led to some most exciting research [6,21,61,62,63,64]. Among many technologies, FISH plays an impressive role. FISH has revolutionized the field of molecular cytogenetics [10]. Unfortunately, many FISH-based cutting-edge platforms, including high-resolution fiber FISH, SKY and M-FISH, and DNA-protein co-detection [11,14,15,18], have yet to be applied to routine clinical analyses. In addition to continually applying current FISH methods in clinic cytogenetics, new combinational FISH methods could be developed with optical genome mapping, DNA-RNA co-detection, Q-FISH, 3-D FISH, and other structural variation platforms [120,121,122]. Encouragingly, highly sensitive FISH, MERFISH (the ‘Multiplexed Error Robust Fluorescence In Situ Hybridization’ method to profile the transcriptome from whole tissue sections to cells and sub-cellular level), and High-Throughput DNA FISH (hiFISH) have been reported [123,124], and more research will soon be followed.

Optical genome mapping is an encouraging example of bridging the detection gap between sequencing and conventional cytogenetics platforms, especially for detecting clonal chromosomal aberrations [120]. This method has already begun to be adopted in cytogenetics laboratories and diagnostic settings. 

## 4. Call to Action

Through this perspective, we call to reevaluate the importance of clinical cytogenetics in the 21st century (rather than replacing cytogenetics with sequencing technologies). The rationale for doing so is not only because the genomic topological information provided by cytogenetics is essential to understanding how the biological system organizes genes but also because the altered karyotypes function as end products of diverse individual molecular mechanisms, which can offer a clinical diagnosis with better prediction. Specifically, with a new framework of the genome architecture theory (GAT), cytogenetics will play an important role in monitoring the somatic evolutionary process, where different stress-induced genomic variations define the dynamic evolutionary phases. Our take-home message is that more effort should be applied to the new frontiers of cytogenetics and cytogenomics. With both novel theoretical and technological innovations, further discoveries and their clinical implication will soon follow. We hope this brief review of both the challenges of current clinical cytogenetics and our personal experience/viewpoints will trigger more discussions in this field, which has huge potential to reshape genomic research and usage in the future [6,42]. Clearly, clinical cytogenetics will play an increasingly important role in personalized medicine.

## Figures and Tables

**Table 1:** Genome Architecture Theory: examples of the rationales, the points, and case studies.

**Rationales for refocusing on cytogenetics:**
Karyotype dynamics and macroevolution [6],
Evolutionary selection unit [28],
Broken promises of the gene-centric theory [28]
Karyotype has better clinical prediction than mutation profiles [43,44,45]
CIN is the common driver which links to diverse gene mutations [46,47]
**Main points of the genome architecture theory (GAT)** [6,28,48]
Karyotype codes system inheritance (a form of inheritance separate from gene-coded inheritance)
Karyotype organizes gene function by defining the network structure [49]
Can explain information creation (new karyotype formation) and preservation (through sexual reproduction) [50,51,52]
System information is linked to many human diseases [40,49]
Two-phased cancer evolution describes varied responses of tumors to stress [53]
Fuzzy inheritance explains missing inheritance [43]
The evolutionary mechanism of cancer unifies diverse molecular mechanisms [22]
‘Game of outliers’ in evolutionary biology [6,47]
The importance of the heterogeneity: reduced specificity of molecular interaction on the gene level [6,53,54]
**Example of the cytogenetic analysis to study cancer evolution:**
Genome chaos: key phase transitions including identifying treatment-induced drug resistance [55,56]
NCCAs as an index of CIN [46]
Polyploid giant cancer cells (PGCCs) link development to evolution [57,58,59,60,61,62,63]
Organismal genomics: karyotype reorganization is the main genomic feature [6]

## Data Availability

Not applicable.
